# Augmented Brain Infiltration and Activation of Leukocytes After Cerebral Ischemia in Type 2 Diabetic Mice

**DOI:** 10.3389/fimmu.2019.02392

**Published:** 2019-10-11

**Authors:** Fang Zhang, Qiuchen Zhao, Yinghua Jiang, Ning Liu, Qiang Liu, Fu-Dong Shi, Junwei Hao, Yun Xu, Eng H. Lo, Xiaoying Wang

**Affiliations:** ^1^Neuroprotection Research Laboratory, Departments of Radiology and Neurology, Massachusetts General Hospital and Harvard Medical School, Boston, MA, United States; ^2^Department of Neurology, Drum Tower Hospital, Medical School of Nanjing University, Nanjing, China; ^3^Department of Neurosurgery, Clinical Neuroscience Research Center, Tulane University School of Medicine, New Orleans, LA, United States; ^4^The Third Affiliated Hospital of Zhengzhou University, Zhengzhou, China; ^5^Barrow Neurological Institute, St. Joseph's Hospital and Medical Center, Phoenix, AZ, United States; ^6^Department of Neurology, Tianjin Neurological Institute, Tianjin Medical University General Hospital, Tianjin, China

**Keywords:** ischemic stroke, distal middle cerebral artery occlusion, diabetes mellitus, immune system, leukocyte mobilization, leukocyte brain infiltration, db/db type 2 diabetes mice

## Abstract

**Background:** Stroke patients with diabetes suffer from higher mortality rate and worsened neurological outcome. However, the responses of immune system to cerebral ischemia in the setting of diabetes remain poorly understood.

**Methods:** In this study, we investigated the temporal profile of leukocyte mobilization and brain infiltration following distal middle cerebral artery occlusion (dMCAO) in db/db mouse model of type 2 diabetes (T2D) and its db/+ normoglycemic controls.

**Results:** We found a significant increase of brain-infiltrating CD4^+^ T cell at day 3 after dMCAO, and a delayed and dramatic increase of brain-infiltrating neutrophils, CD4^+^ T cells, CD8^+^ T cells, and B cells at day 7 after dMCAO in db/db mice vs. db/+ controls. Leukocyte subsets in the circulation and spleen were also measured, however, there is no significant difference between non-diabetic and diabetic groups. Furthermore, we identified an increased expression of activation marker CD69 in brain-infiltrating neutrophils, CD4^+^ T and CD8^+^ T cells, and IFN-γ in brain-infiltrating CD4^+^ T cells in db/db mice at day 7 after dMCAO.

**Conclusions:** These findings for the first time demonstrate that cerebral ischemia induces a delayed and sustained augmentation of brain infiltration and activation of neutrophils and lymphocytes in type 2 diabetic mice and these altered immune responses might contribute to the severer brain tissue damage and worse neurological outcomes of diabetes stroke, which warrants further investigation.

## Introduction

Stroke is a leading cause of death and long-term disability accompanied by a major economic and healthcare burden. Among the prominent risk factors of stroke, diabetes mellitus (DM) has been linked to higher mortality rate and worsened neurological outcome in stroke patients (1–3). Approximately 30% of stroke patients are diabetic (4, 5), however, the underlying mechanisms responsible for the increased post-ischemic brain injury in subset of stroke patients remain poorly understood.

Nearly 90% of DM patients suffer from type 2 diabetes (T2D). Emerging evidence suggests that immune and inflammatory response is a critical driver in the pathogenesis of T2D, including obesity-related insulin resistance, impaired insulin secretion, and diabetes-related vascular complications (6–14). Given that inflammation is a key participant in brain injury and recovery after ischemia, and that the immune system and its responses to injury is profoundly altered in diabetes, how the immune system respond to cerebral ischemia in the setting of diabetes remains a fundamental yet unanswered question.

The leptin receptor deficient db/db mice are the most commonly used T2D rodent models in study of diabetes pathophysiology and complications including ischemic stroke (15–18). It has been reported that after focal cerebral ischemia, diabetic db/db mice presented confounding pathological features, including metabolic dysregulation, systemic, and vascular inflammation, aggravated blood-brain barrier integrity disruption and pro-inflammatory response, white matter integrity loss, severer brain damage, and worse neurological deficits (19, 20). These pathological outcomes closely mimic clinical observations (21, 22). In the same distal middle cerebral artery occlusion (dMCAO) stroke model of db/db mice, our previous study showed that compared to the non-hyperglycemic genetic control mice, T2D db/db mice had sustained hyperglycemia after stroke, elevated blood HbA1c level, hyperinsulinemia, and lowered serum adiponectin level (20). Importantly, we also found there are a larger infarct and an aggravated pro-inflammatory response including increased mRNA expression of pro-inflammatory cytokines and elevated M1-like pro-inflammatory microglia/macrophage activation in the T2D db/db mouse brain after dMCAO (20). To explore the potential role of infiltrated leukocytes in the ischemic brain inflammation process of T2D stroke, for the first step, in this study we investigated the temporal profile of leukocyte mobilization and infiltration in adult db/db mice following dMCAO.

## Materials and Methods

All experiments were conducted in accordance with the standards and procedures of the American Council on Animal Care and Use Committee of Massachusetts General Hospital Neurological Institute. The study design, power calculations, experiments conduct, statistical analyses, and results reporting all fulfilled the ARRIVE guidelines (23).

### dMCAO Model

The db/db and db/+ mice (12 weeks) were purchased from Charles River Laboratories. Mice were maintained in Allentown individually ventilated caging with acidified water in bottles and rodent chow fed *ad libitum*. Lights are on a 12 h/12 h light/dark cycle, ON at 7:00 a.m., OFF at 7:00 p.m. Room temperature is maintained between 22 and 30°C and humidity is maintained between 30 and 70%. Mice were housed in pathogen-free condition at the Massachusetts General Hospital Neurological Institute. Male db/db and db/+ mice were used in this study. Anesthesia was induced by 2.5% isoflurane and maintained with 1.5% isoflurane during surgery. Core body temperatures were monitored with a rectal probe. The distal middle cerebral artery (dMCA) was exposed and cauterized above the rhinal fissure. The bilateral common carotid arteries (CCAs) were occluded for 90 min and then released, whereas the dMCA remained occluded. Animals received intensive care and continuous monitoring until they were capable of functioning normally (24). There was no mortality in each group of present study. Body weight and blood glucose levels have no statistic difference between sham and dMCAO stroke groups neither in db/db nor db/+ group. As reported previously, there are significant differences of body weight and blood glucose levels between db/db and db/+ control mice (Supplementary Figure 1) (20).

### Flow Cytometry

Single-cell suspensions were prepared from spleen, blood or brain tissues of sham or dMCAO db/db and db/+ mice at day 3 and 7. To collect cells from brain, brain tissues were grinded and homogenized with 40 mm nylon cell strainers in PBS. Cell suspensions were centrifuged at 2,000 rpm for 5 min, and cell pellets were collected. Thereafter, 5 ml of 30% Percoll solution was used to resuspend the cell pellet. The gradient was centrifuged at 2,000 rpm for 30 min at room temperature. Cell pellets were collected for antibody staining. Then these cells were stained with fluorochrome conjugated antibodies. All antibodies were purchased from BD Bioscience (Franklin lakes, NJ, USA) or Biolegend (San Diego, CA, USA). Antibodies were directly labeled with one of the following fluorescent tags: fluorescein isothiocyanate (FITC), phycoerythrin (PE), allophycocyanin (APC), PerCP-Cy5.5, or PE-Cy7. The following antibody to mouse antigens were used: CD3, CD4, CD8, CD19, CD45, CD11b, F4/80, Ly6G, Interferon gamma (IFN-γ) (XMG1.2), CD69 (H1.2F3), CD86 (GL1), CD206 (MR5D3). Antibodies staining were performed according to their instructions, additional cell fixation and permeabilization were needed for intracellular antigens staining. Cell surface phenotype and intracellular cytokine expression were performed on a FACS FORTESSA flow cytometer (BD Bioscience, Franklin lakes, NJ, USA). Data were analyzed with Flow Jo software version 7.6.1 (Flow J, LLC, Ashland, OR, USA).

### Immunohistochemistry

Paraffin-embedded tissue sections at thickness of 8 μm were used in this study. Coronal sections were prepared from 1 mm behind the bregma. For immunostaining, the following primary antibodies were used: anti-CD4, C-Terminal antibody produced in rabbit (1: 200, SAB4503583, Sigma-Aldrich), Purified Rat Anti- Mouse CD8a (1: 200, 550281, BD Biosciences), Alexa Fluor® 647 anti-mouse CD19 Antibody (1: 100, 550281, BioLegend), Purified Rat Anti-Mouse Ly-6G (1: 200, 550291, BD Biosciences), Alexa Fluor® 488 anti-mouse CD45.2 Antibody (1: 100, 109815, BioLegend). Primary antibodies were incubated at 4°C overnight, followed by incubation with species-specific Alexa Fluor (488 and 594)-conjugated secondary antibodies for 1 h. Pictures were acquired with a Nikon Eclipse T300 fluorescence microscope and analyzed using Image Pro Plus (Media Cybernetics, Inc. Rockville, MD). For cell counting, positive cell numbers were counted in every tenth tissue section through the entire tissue block (25–27).

### Quantitative Real-Time PCR

Brain tissues of peripheral infarct were obtained from mice 1 and 3 days after distal MCAO model. Total RNA was extracted and reverse-transcribed using RNeasy Lipid Tissue Mini Kit (Qiagen) and QuantiTect reverse transcription system (Qiagen) according to the manufacturer's instructions. Real-time polymerase chain reaction (PCR) was performed on an ABI 7500 Fast Real-Time PCR system using Taqman gene expression assays for CX3CL1 (Mm00436454_m1), CXCL12 (Mm00445553_m1), CCL2 (Mm00441242_m1), CCL9 (Mm00441260_m1), and housekeeping gene B2M (Mm00437762_m1) (Applied Biosystems, USA). Reactions were performed in duplicate according to the manufacturer's instructions. Relative expression levels were measured with the 2^−ΔΔCt^ method.

### Statistical Analysis

Based on our publications (20, 27) and power analysis, at least six biological replicates were used for each biochemical and histological analysis, whereas a sample size of 8 per group was used for assessments of leucocytes number and function. Sample size per experimental model was determined a priori by performing a power calculation with G^*^Power (3.1) software. D'Agostino and Pearson omnibus normality test were performed to determine normal distribution. All surgery and histology measures were performed by researchers who were blinded with respect to the different groups. All results were expressed as mean ± s.e.m. Statistical analysis was performed using Graphpad prism 7.6.1 (Graphpad Software Inc., San Diego). Two-tailed unpaired *t*-test was used for comparison between two groups. One-way ANOVA followed by Tukey *post-hoc* test to compare three or more groups.

## Results

### Augmented Brain Leukocyte Infiltration in db/db Mice Following Cerebral Ischemia

**Figure 1:**
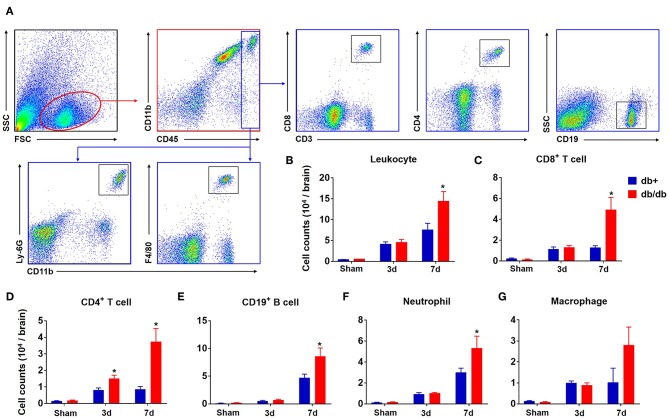
Augmented brain infiltration of leukocyte subsets in db/db mice subjected to dMCAO assessed by flow cytometry. Groups of db/db or db/+ mice were subjected to sham or dMCAO surgery. Single-cell suspensions were prepared from brain tissues of indicated groups of mice. **(A)** Gating strategy of peripheral leukocytes (CD45^+^), including macrophages (CD45^high^CD11b^+^ F4/80^+^, Mφ), neutrophils (CD45^high^ CD11b^+^ Ly-6G^+^), CD4^+^ T (CD45^high^ CD3^+^ CD4^+^), CD8^+^ T (CD45^high^ CD3^+^ CD8^+^), and B (CD45^high^ CD19^+^) cells in the ischemic brain at day 3 and day 7 after dMCAO. **(B–G)** Quantification of brain-infiltrating lymphocytes, macrophages and neutrophils from sham and distal MCAO db/+ and db/db mice at indicated time points after ischemia. Data are expressed as mean ± s.e.m. *p < 0.05: db/+ vs. db/db at the same time point, *n* = 8 per group.

**Figure 2:**
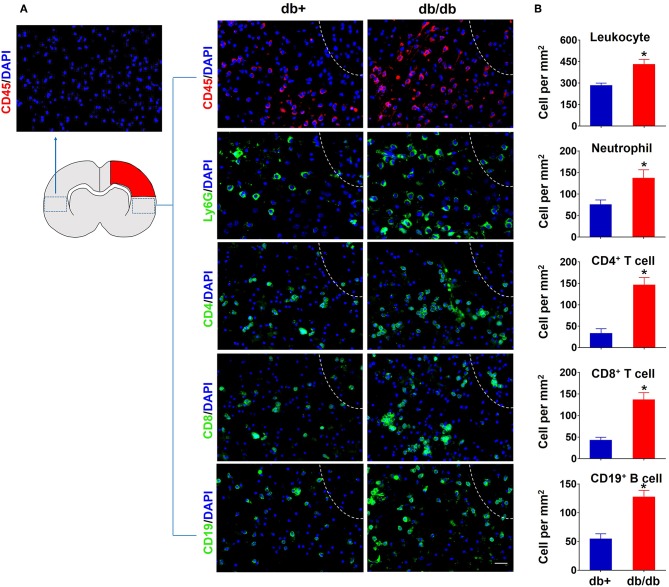
Accumulation of brain-infiltrating leukocyte subsets in the ischemic brain of db/db mice subjected to dMCAO assessed by immune staining. **(A)** At 7 days after dMCAO, increased counts of CD45^+^ leucocytes, CD4^+^ T, CD8^+^ T, CD19^+^ B cells, and Ly-6G^+^ neutrophils were seen in the peri-infarct region of brain sections from db/db mice vs. db/+ controls. The right side of white lines represents infarct area. Scale bars: 50 μm. **(B)** Quantification of brain-infiltrating immune cell subsets in db/+ and db/db mice subjected to dMCAO at day 7 after ischemia. Data are expressed as mean ± s.e.m. *p < 0.05: db/+ vs. db/db, *n* = 8 per group.

To characterize the profile of immune responses in diabetic stroke, we first measured the counts of brain-infiltrating leukocytes in db/db and db/+ mice subjected to dMCAO using flow cytometry. The gating strategy of immune cell subsets is shown in Figure 1A. At 3 days after ischemia, the total numbers of leucocytes (CD45^high^), macrophages (CD11b^+^CD45^high^F4/80^+^), neutrophils (CD11b^+^CD45^high^Ly-6G^+^), B cells (CD19^+^), or CD8^+^ T cells (CD3^+^CD8^+^) were significantly increased in the ischemic brains of both db/db and db/+ mice (Figures 1B,C,E–G). Interestingly, db/db mice had significantly higher elevation of increased infiltrating CD4^+^ T cells (CD3^+^CD4^+^) at 3 days after dMCAO compared to db/+ mice (Figure 1D). Importantly, at day 7 after dMCAO, significantly increased numbers of infiltrating leucocyte subsets, including CD4^+^ T cells, CD8^+^ T cells, B cells, and neutrophils, were observed in db/db mice as compared to db/+ mice. Next, immunostaining was performed to verify our flow cytometry findings. At day 3 after dMCAO, an increase of infiltrating CD4^+^ T cells was seen in the peri-infarct area of db/db mice. Similarly, augmented infiltration of CD4^+^ T cells, CD8^+^ T cells, B cells, and neutrophils was found in db/db mice at day 7 after dMCAO (Figures 2A,B). Together, these data demonstrate that the augmented infiltration of leukocytes in the ischemic brain of db/db mice involves a significant elevation of CD4^+^ T cells at day 3, and the delayed and sustained elevation of leukocytes up to 7 days after dMCAO.

### Leukocyte Subsets in the Circulation and Spleen of db/db Mice vs. db/+ Controls After dMCAO

**Figure 3:**
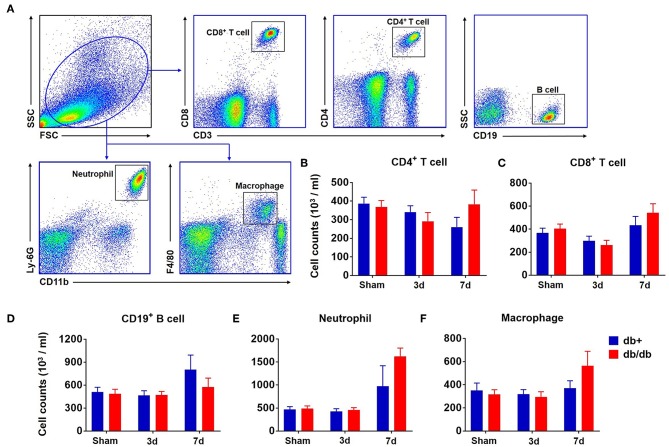
Counts of circulating leucocytes in db/db and db/+ mice following brain ischemia. Groups of db/db and db/+ mice were subjected to sham or dMCAO surgery. The counts of circulating leucocyte subsets were measured by flow cytometry in indicated groups of mice. **(A)** Gating strategy of macrophages (CD11b^+^ F4/80^+^, Mφ), neutrophils (CD11b^+^ Ly-6G^+^), CD4^+^ T (CD3^+^ CD4^+^), CD8^+^ T (CD3^+^ CD8^+^), and B (CD19^+^) cells in the blood at day 3 and 7 after dMCAO. **(B)** Numbers of CD4^+^ T cells (CD3^+^CD4^+^), **(C)** CD8^+^ T cells (CD3^+^CD8^+^), **(D)** CD19^+^ B cells (CD19^+^), **(E)** neutrophils (CD11b^+^Ly-6G^+^), or **(F)** macrophages (CD11b^+^F4/80^+^) in the blood of indicated groups of mice. Data are expressed as mean ± s.e.m, *n* = 8 per group.

**Figure 4:**
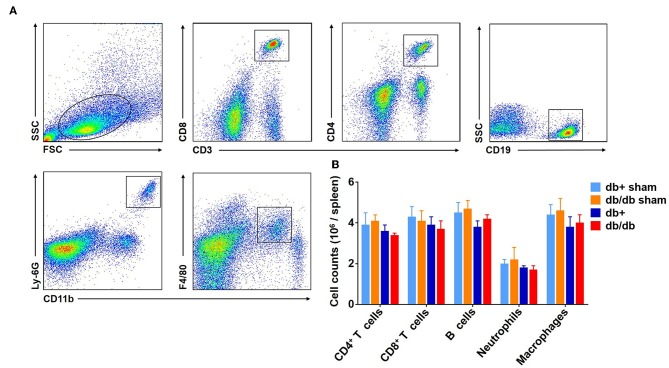
Counts of splenic leucocytes in db/db and db/+ mice following brain ischemia. The counts of splenic leucocyte subsets were measured at 3 days after dMCAO in groups of db/db and db/+ mice by flow cytometry **(A)**. Gating strategy of macrophages (CD11b^+^ F4/80^+^, Mφ), neutrophils (CD11b^+^ Ly-6G^+^), CD4^+^ T (CD3^+^ CD4^+^), CD8^+^ T (CD3^+^ CD8^+^), and B (CD19^+^) cells in the spleen at day 3 after dMCAO. **(B)** Quantification of splenic lymphocytes, macrophages and neutrophils in indicated groups of db/+ and db/db mice. Data are expressed as mean ± s.e.m, *n* = 8 per group.

In addition to the brain, we also measured the counts of macrophages, neutrophils, CD4^+^ T, CD8^+^ T, and B cells in the blood (Figure 3A). Our results showed that there was no significant difference in the numbers of CD4^+^ T cells, CD8^+^ T cells, B cells, neutrophils, and macrophages in the blood of db/db mice vs. db/+ controls at day 3 and 7 after dMCAO (Figures 3B–F). Similarly, no significant alterations of these leukocyte subsets were seen in the spleen of db/db mice vs. db/+ controls (Figures 4A,B). These data suggest that except elevated and sustained brain infiltration, peripheral inflammatory cell mobilization after ischemic stroke might not be significantly altered by DM, at least in the adult db/db type 2 male mice after dMCAO.

### Upregulation of CD69 and IFN-γ in Brain-Infiltrating Leucocytes of db/db Mice Subjected to dMCAO

**Figure 5:**
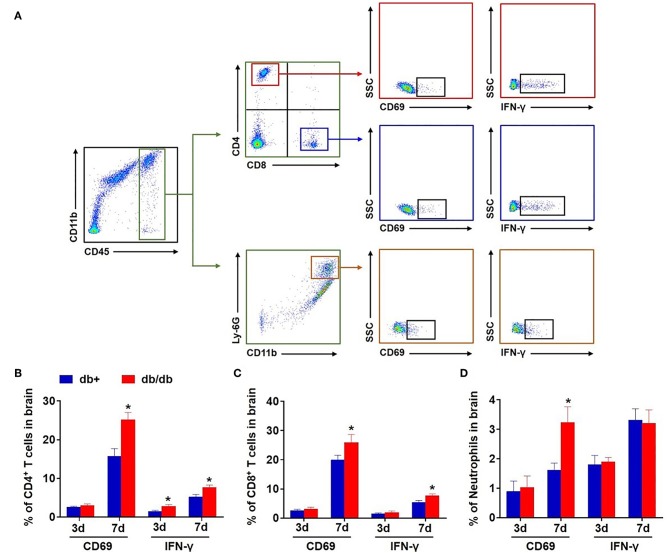
Upregulation of CD69 and IFN-γ in brain-infiltrating T cells and neutrophils in db/db mice subjected to dMCAO. **(A)** Gating strategy of brain-infiltrating CD4^+^ T, CD8^+^ T cells, and neutrophils expressing CD69 and IFN-γ after dMCAO. The counts of CD4^+^ T **(B)**, CD8^+^ T cells **(C)**, and neutrophils **(D)** expressing CD69 and IFN-γ in groups of db/+ and db/db mice at indicated time points after dMCAO. Mean ± s.e.m.; *p < 0.05: db/+ vs. db/db at the same time point, *n* = 8 per group.

Next, we examined the expression of the leukocyte activation marker CD69 and pro-inflammatory cytokine IFN-γ in brain-infiltrating leukocyte subsets after dMCAO. We found an upregulation of CD69 in brain-infiltrating CD4^+^ T, CD8^+^ T cells, and neutrophils in db/db mice accompanied by an increased expression of IFN-γ at day 7 after dMCAO (Figures 5A–D).

**Figure 6:**
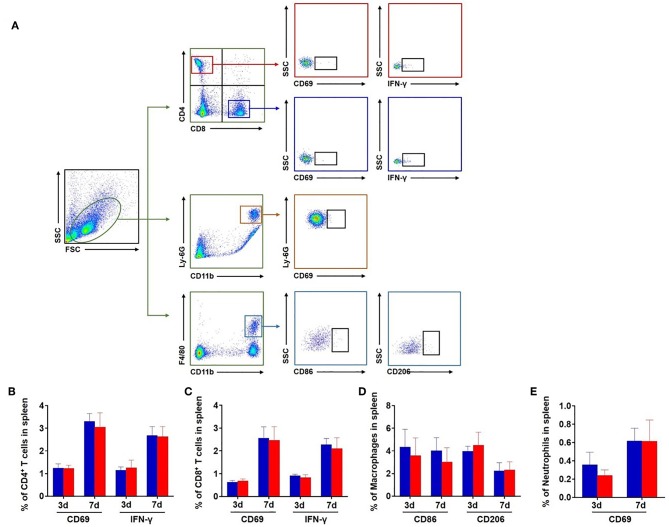
Expression of CD69 and IFN-γ in splenic leucocytes of db/db and db/+ mice after brain ischemia. **(A)** Gating strategy of CD4^+^ T, CD8^+^ T cells, and neutrophils expressing CD69 and IFN-γ and macrophages expressing CD86 and CD206 in the spleen after dMCAO. The counts of CD4^+^ T **(B)**, CD8^+^ T cells **(C)**, and neutrophils **(D)** expressing CD69 and IFN-γ in the spleen of db/+ and db/db mice at 3 and 7 days after ischemia. The counts of macrophages **(E)** expressing CD86 and CD206 in the spleen of db/+ and db/db mice at 3 and 7 days after ischemia. Mean ± s.e.m. *n* = 8 per group.

In contrast, the expression of CD69 and IFN-γ was not significantly altered in splenic CD4^+^ T, CD8^+^ T cells, and neutrophils in db/db mice vs. db/+ controls following dMCAO (Figures 6A–D). The expression of CD86 and CD206 was also not altered in splenic macrophages (Figure 6E). Together, these results suggest that the augmentation of leukocyte response primarily occurs in the ischemic brain but not in the periphery in db/db mice after dMCAO.

## Discussion

After permanent focal ischemia, diabetic db/db mice presented confounding pathological features, including metabolic dysregulation, more severe brain damage, and neurological impairment, especially aggravated pro-inflammatory response and white matter integrity loss (16, 19, 20). In this study, we provide the first definitive evidence of augmented brain infiltration of neutrophils and lymphocytes in type 2 diabetic mice following cerebral ischemia. We found a delayed and sustained augmentation of brain infiltration of neutrophils and lymphocytes in type 2 diabetic mice up to 7 days after focal cerebral ischemia. There was also a significant upregulation of activation marker in these infiltrated cells. In contrast, the counts of leukocytes were not significantly altered in the periphery.

The finding of augmented brain infiltration of leukocytes in diabetic mice following cerebral ischemia indicates that these cells may contribute to exacerbated ischemic brain injury in diabetes. This speculation is supported by our previous publications showing that blockade of leukocytes homing into ischemic brain has neuroprotective and anti-inflammatory benefits (28, 29). As these infiltrating leukocytes can boost the brain inflammatory environment by producing various effector molecules or inflammatory mediators, they may amplify the pre-existing cerebral microvascular injury in diabetes to augment brain edema and neural injury after ischemia (30). Different than the augmented infiltration of leukocytes in the brain of diabetic stroke mice, the leukocyte responses in the peripheral compartment were relatively unaltered. These findings together suggest that diabetes-associated alterations of leukocyte responses predominantly occur in the ischemic brain. In humans, obesity and T2D induce the expansion of pro-inflammatory T cells such as CD4 (Th1, Th17) and CD8 populations, whereas innate T cells such as invariant natural killer T cells and mucosal-associated invariant T cells were found reduced (31). Peripheral blood monocytes from T2D patients were found constitutively activated (32). T2D has also been associated with changes in neutrophil function including impaired bacterial phagocytosis and killing activity (33). Although the brain infiltration profiles of leukocytes have not been investigated in diabetic patients, the similarity and discrepancy of peripheral immune responses in diabetic patients and db/db mice might involve the complicated effects of diabetes on the immune system and warrant further investigation.

The early upregulation of chemokines in the brain of diabetic mice after ischemia suggests that the augmented brain infiltration of leukocytes in diabetic stroke may involve the upregulation of chemokines in the brain. Because the activity of leukocytes is largely determined by the environment in which they reside, it is logic to infer that diabetes may alter the brain environment that foster the cues to recruit peripheral leukocyte subsets after stroke. Once enter the ischemic brain, infiltrating leukocytes will be exposed to additional factors that are different than those in the peripheral compartment. This notion is supported by our observation of upregulation of activation marker CD69 and IFN-γ in brain-infiltrating leukocytes in diabetic stroke. Future studies will be necessary to identify these unique brain-derived factors that may govern the phenotype and function of infiltrating leukocytes in diabetic stroke.

Preclinical and clinical studies indicate that modulation of the immune system attenuates ischemic brain injury (34, 35). These immune-driven effects may have broad implications for both stroke and diabetes, because inflammation has been established as a link between vascular complications in diabetes and cerebrovascular disease risk. In this regard, anti-inflammatory medications targeting components of the immune system have shown beneficial effects on glycemia, β-cell function, and insulin resistance (36). For example, independent clinical studies conducted with an IL-1 receptor antagonist (anakinra) or IL-1β-specific antibody (canakinumab) have demonstrated beneficial effects on metabolic parameters including decreased HbA1c and enhanced insulin sensitivity and β-cell secretory function, with concomitant improvement in inflammatory markers in diabetes (37, 38) and significantly reduced recurrent cardiovascular events (39). Thus, a combination therapy that can curtail multiple pathophysiological mechanisms including inflammation and hyperglycemia might be a good choice for preventing exacerbated brain damage in diabetic stroke patients. In this study, we found that the immune response of ischemic stroke combined with diabetes is very different from simple ischemic stroke. In terms of future anti-stroke therapeutics, an important goal is to realize the theory of precision medicine, which is based on the concept that the etiology is not the same for all patients. For example, the quantitative contribution of inflammation and immune response will differ between subset patients, such as diabetic stroke complication. For the first step, we investigated the temporal profile of leukocyte mobilization and infiltration in adult db/db male mice following dMCAO. An important future goal is to gain a clear understanding of the underlying molecular mechanisms for the different inflammatory responses of diabetic stroke, which would help in development of specific and effective anti-diabetic stroke therapies.

We are aware that there are several limitations in this study. First, we used C57BLKS-Leprdb T2D mice (db/db T2D mice, Jackson Lab), the leptin receptor mutation does not reflect disease etiology in humans, although this model provides us insight into glucose metabolism and identified novel pathways of its complications (40). However, there are variable pathogenic mechanisms between different T2D animal models, investigation of inflammatory response in other diabetes animal models with ischemic stroke should be pursued in the future. Second, in this pilot study, we characterized the temporal profile of leukocyte mobilization and brain infiltration in T2D stroke mice. This servers as the first step for our long-term goal to understand whether and how infiltrating leukocytes contribute to exacerbated brain infarction in process of T2D stroke. Although augmented neuroinflammation has been linked to worsened neurological outcome in T2D stroke, the contribution from specific components of immune system to T2D-related brain pathology such as microvascular dysfunction and increased BBB permeability remains elusive. In this regard, future studies are warranted to unveil the precise role of immune components in these pathological processes of T2D stroke. Third, we found highly elevated CD4^+^ T cells in the early stage, delayed and sustained inflammatory cells brain infiltration in T2D stroke mice, but their pathological roles and underlying mechanism in modulating the different inflammatory response needs to be defined in the future. Fourth, although an altered macrophage response was observed in diabetic mice, we did not investigate polarization of macrophages. Considering the central role of macrophage in tissue injury and repair, future studies are required to further investigate macrophage responses in this model. Last, this study was proposed as a proof-of-concept investigation. Indeed, there are multiple pathological factors that dynamically and interactively participate in inflammation-associated T2D stroke brain damage evolution and recovery processes (19), which require further investigation. In addition, although a larger infarct volume in db/db mice might be a contributor to increased brain infiltration of leukocyte, it is also noteworthy that the increase of brain-infiltrating total leukocytes, neutrophils, macrophages, CD8^+^ T cells, B cells and lymphocytes did not occur at day 3 after stroke. A preferential increase of CD4^+^ T cells at day 3 after stroke in db/db mice suggests possible involvement of environmental cues in diabetic brain that elicit an early CD4^+^ T cell response. We also acknowledge the possible limitation of insufficient sample size or relatively large variations, and will extend our studies in the future.

## Conclusions

In summary, experimental results from this study have demonstrated a delayed and sustained augmentation of brain infiltration and activation of neutrophils and lymphocytesafter cerebral ischemia in type 2 diabetic mice. The potential contribution of these altered immune responses to the severer brain damage in diabetes stroke awaits further investigation.

## Data Availability Statement

All datasets generated for this study are included in the manuscript/Supplementary Files.

## Ethics Statement

All animal experiments were performed in accordance with the ARRIVE (Animal Research: Reporting *in vivo* Experiments) guidelines. All procedures were approved by Animal Care and Use Committees of the Massachusetts General Hospital Neurological Institute.

## Author Contributions

FZ and QZ acquired, analyzed, and interpreted the data and drafted the manuscript. YJ and NL made the stroke mouse model and brain tissue dissection. QL, EL, JH, F-DS, and YX interpreted the data and drafted and edited the manuscript. XW and FZ formulated the study concept, designed the study, and edited the manuscript.

### Conflict of Interest

The authors declare that the research was conducted in the absence of any commercial or financial relationships that could be construed as a potential conflict of interest.

## Abbreviations

dMCAO: distal middle cerebral artery occlusion
T2D: type 2 diabetes
DM: diabetes mellitus.
