# Supplementary material for: Augmented Brain Infiltration and Activation of Leukocytes After Cerebral Ischemia in Type 2 Diabetic Mice
Source: Front Immunol. 2019 Oct 11;10:2392. doi: 10.3389/fimmu.2019.02392 (PMC6797587; doi:10.3389/fimmu.2019.02392)
Supplement: Supplementary file 1 [file Data_Sheet_1.docx]

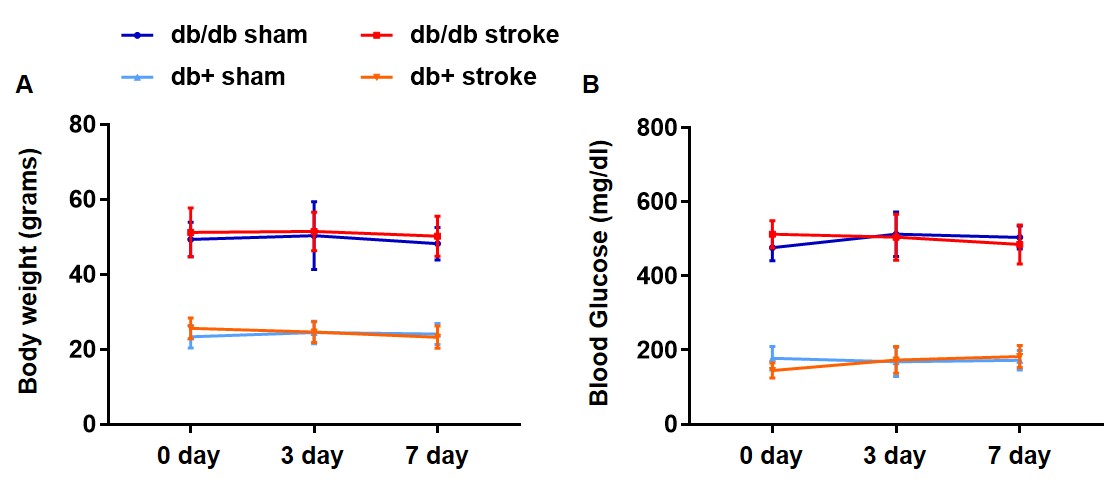


**Supplementary Figure 1.** Body weight and blood glucose levels. We tested the body weight (A) and blood glucose levels (B) before and 3 days, 7 days after stroke. There is no statistic difference between sham and dMCAO stroke groups neither in db/db nor db/+ group. As reported previously, there are significant differences of body weight and blood glucose levels between db/db and db/+ control mice ^1^.

Reference

1. Jiang Y, Liu N, Wang Q, et al. Endocrine Regulator rFGF21 (Recombinant Human Fibroblast Growth Factor 21) Improves Neurological Outcomes Following Focal Ischemic Stroke of Type 2 Diabetes Mellitus Male Mice. *Stroke*. 2018; 49: 3039-49.
